# Immersive Reality Experience Technology for Reducing Social Isolation and Improving Social Connectedness and Well-being of Children and Young People Who Are Hospitalized: Open Trial

**DOI:** 10.2196/29164

**Published:** 2022-03-25

**Authors:** Hiran Thabrew, Laura A Chubb, Harshali Kumar, Christa Fouché

**Affiliations:** 1 The Werry Centre, Department of Psychological Medicine Faculty of Medical and Health Sciences University of Auckland Auckland New Zealand; 2 Faculty of Education and Social Work The University of Auckland Auckland New Zealand

**Keywords:** immersive reality experiences, social isolation, hospitalized children, well-being, social connectedness, immersive reality, virtual reality, serious games, pediatrics, mental health, isolation, hospitalized patients, adolescents

## Abstract

**Background:**

Children and young people who are hospitalized can feel disconnected from their peers and families, which can, in turn, predispose them to psychological problems, including anxiety and depression. Immersive reality experience technology, recently developed by the New Zealand Patience Project Charitable Trust, may help to overcome these issues. Immersive reality experience technology uses immersive 360° live streaming and a virtual reality headset to enable children and young people who are hospitalized to connect through cameras located in either their school or home environment and via SMS text messaging with a designated buddy.

**Objective:**

This trial aims to expand qualitative findings from a previous smaller *proof of concept* trial to ascertain the views of New Zealand children and young people who are hospitalized, their caregivers, and teachers regarding immersive reality experience technology and quantitatively evaluate the effectiveness of immersive reality experience technology in reducing social isolation and improving social connectedness and well-being using validated outcome measures.

**Methods:**

An open trial of immersive reality experience technology was conducted between December 2019 and December 2020 for which 19 New Zealand children and young people aged 13 to 18 years who had been hospitalized at Starship Hospital—a specialist pediatric hospital in Auckland—for at least 2 weeks were recruited. All young people completed the Short Warwick–Edinburgh Mental Well-Being Scale, an abbreviated version of the Social Connectedness Scale, and the Social Inclusion Scale at baseline. Of the 19 participants, 10 (53%) used immersive reality experience technology as often as they wished over a 6-week period and completed postintervention measures. Semistructured interviews with a subset of the 10 young people, 4 caregivers, and 6 teachers were conducted immediately after the intervention.

**Results:**

Participants reported improvements in social inclusion (mean change 3.9, SD 2.8; *P*=.06), social connectedness (mean change 14.2, SD 10.0; *P*=.002), and well-being (mean change 5.7, SD 4.0; *P*=.001). Key themes from interviews with participants, caregivers, and teachers were the importance of support for using immersive reality experience technology, connecting versus connectedness, choice and connection, and the value of setting it up and getting it right. Recommendations for improving connectedness via immersive reality experience and related technologies were also provided.

**Conclusions:**

Immersive reality experience technology can improve the social inclusion, social connectedness, and well-being of New Zealand children and young people who are hospitalized. With some technological modifications and simplified implementation, immersive reality experience and related technology could become part of standard care and support children and young people who are hospitalized in New Zealand and elsewhere to sustain family and peer cohesion, experience fewer psychological problems, and more easily return to normal life following the completion of treatment.

**Trial Registration:**

Australian New Zealand Clinical Trials Network Registry ACTRN12619000252112; https://www.anzctr.org.au/Trial/Registration/TrialReview.aspx?id=376837&isReview=true

## Introduction

### Background

Between 10% and 12% of children and young people worldwide, and up to 22% of New Zealand Māori taitamariki (children and young people), experience long-term physical conditions (chronic illnesses) such as cancer, diabetes, and cystic fibrosis [1]. Many spend weeks or months in hospitals, disconnected from their wider families, peers, and schools [2]. Social disruption and exclusion may be related to psychological problems, including anxiety and depression, and may be associated with reduced academic achievement [3-5]. Conversely, social inclusion during treatment may be associated with improved psychosocial functioning [6]. Psychosocial support offered to children and young people who are hospitalized varies widely [7]. For example, many rural institutions or those in lower income countries offer minimal formal support, whereas specialist pediatric centers in others have access to multiple resources (eg, on-site consultation liaison, mental health teams, play specialists, and volunteer organizations). Despite the support available in more developed countries, resources are usually focused on supporting medical treatment rather than improving social connections [8].

Over the past few decades, advances in technology have led to the development of a wide array of eHealth interventions, including websites, self-help apps, health games, and devices that provide immersive reality experiences [9]. A number of these were developed for, or trialed with, children and young people with long-term physical conditions [10]. Immersive reality experience technology engages users in an alternate, real environment, virtual environment, or a combination of real and virtual environments [11]. Immersive reality experience technology has been shown to reduce psychological stress and improve mental health in adults [12,13], children with disabilities [14], and older adults [15]. In some cases, immersive reality experience technology has been shown to enhance engagement in schoolwork and improve emotional well-being in children and young people with long-term physical conditions [14-17]. However, factors affecting the use of immersive reality experience technology by this group, such as hardware issues, privacy concerns, and the impact of health status and hospitalization, are not well-described in the literature.

In 2018, the Patience Project, a New Zealand charitable organization headed by one of the members of our research group (BM), developed a virtual reality (VR) environment–based system of immersive reality experience technology to connect children who are hospitalized with peers at home and school. Two-way communication was possible through the young person using the immersive reality experience technology texting a designated buddy in their school environment to ask questions to the teacher on behalf of the absent young person or strike up a conversation with their peers. This was the first intervention of its kind in New Zealand. A formative assessment of the Patience Project was conducted with 15 participants in 2018 [18] and aimed at developing an understanding of the perceived strengths and weaknesses of the project to inform future directions. A total of 5 children receiving oncological treatment at Starship Hospital, a tertiary pediatric hospital in Auckland, New Zealand, along with their teachers and caregivers, comprised the sample. Despite the small sample, the formative assessment elicited valuable information about young people who experience social disruption and the potential for immersive technology to facilitate and sustain connectedness to familiar environments and to peers. This exploratory investigation supported the viability of undertaking a more formal open trial described in this paper.

### Objectives

This trial was conceptualized by two authors (HT and CF) with the following aims:

1. To investigate the acceptability of immersive reality experience technology for children who are hospitalized, families, and school staff
2. To examine the effectiveness of this technology in improving social connectedness, reducing social isolation, and improving the well-being of children who are hospitalized
3. To provide information regarding the feasibility of a more definitive randomized controlled trial (RCT)

## Methods

### Recruitment and Sample

A mixed methods trial design was used. We aimed to recruit a convenience sample of up to 40 children and young people admitted to Starship Hospital, a tertiary pediatric hospital in Auckland, New Zealand, between December 2019 and December 2020. However, because of the COVID-19 pandemic and its potential risks to the young people in our sample who already have compromised immune systems, we were unable to recruit all 40 potential participants for 6 months between March and September 2020. This led to the final recruitment of 19 young people to trial the immersive reality experience technology, 10 (53%) of whom felt well enough to complete the intervention. In addition to the study participants, we also aimed to recruit up to 10 caregivers and 10 school staff who supported the young person’s participation in the classroom to take part in semistructured interviews after the intervention. Of these 10 young people, 4 (40%) young people and their respective caregivers, together with 7 teachers, provided in-depth feedback. Fortunately, this number was sufficient for us to obtain rich qualitative data regarding the acceptability of immersive reality experience technology and some quantitative data regarding its effectiveness.

The participants were provided with information about the trial via their clinical teams. Participating teachers were actively recruited by a research assistant. All other participants learned about the project through waiting room conversations at the hospital with a physician or nurse or, in one case, a television advertisement. The required inclusion criteria for young people to participate in this trial were as follows: (1) aged 13 to 18 years, (2) presence of any medical condition, and (3) admitted to Starship Hospital for more than a 2-week period or intermittently over a 6-month time frame. Three exclusion criteria were set as follows: (1) children aged <13 years or adults aged >18 years, (2) individuals with a physical or mental health issue that prevented exposure to immersive reality experience technology (eg, severe seizures), and (3) those not able to provide informed consent (or assent with caregiver consent). All participants received a US $25 gift voucher for their participation.

A total of 19 young people were recruited via their usual clinicians at Starship Hospital, of which 10 (53%) used the intervention. Of the 9 young people who did not use immersive reality experience technology, 2 (22%) reportedly found it too difficult to use, 1 (11%) was too unwell for the duration of the trial, 1 (11%) left the hospital sooner than expected, 1 (11%) did not provide a reason, and the schools of 4 (44%) other young people declined to allow immersive reality experience technology to be used in their classrooms despite ethics committee approval of the project and personal explanation by a research assistant.

All 19 young people completed preintervention questionnaires at the time of recruitment, and the 10 young people who completed the intervention filled out postintervention questionnaires. Only 40% (4/10) of young people took part in semistructured interviews; thus, their data were analyzed alongside those of caregivers and teachers. Characteristics of all the recruited young people are further described in Table 1. To maximize confidentiality, no data were collected regarding the type of long-term physical condition or conditions participants were experiencing.

**Table 1:** Characteristics of participants.

Characteristics		All participants				Participants who completed the intervention				Participants who completed interviews		
		Young people (n=19)	Caregivers (n=4)	Teachers (n=6)	Young people (n=10)		Caregivers	Teachers	Young people (n=4)		Caregivers (n=4)	Teachers (n=6)
Age (years), mean (SD; range)		14.3 (1.3; 12-17)	N/A^a^	N/A	14.2 (1.0; 13-16)		N/A	N/A	14.3 (1.0; 13-15)		N/R^b^	N/R
**Sex, n (%)**												
	Male	9 (45)	0 (0)	3 (50)	5 (50)		N/A	N/A	1 (25)		0 (0)	3 (50)
	Female	10 (50)	4 (100)	3 (50)	5 (50)		N/A	N/A	3 (75)		4 (100)	(50)
**Ethnicity, n (%)**												
	New Zealand European	5 (26)	3 (75)	6 (100)	3 (30)		N/A	N/A	3 (75)		3 (75)	6 (100)
	New Zealand Māori	5 (26)	0 (0)	0 (0)	1 (10)		N/A	N/A	0 (0)		0 (0)	0 (0)
	Pacific Islander	5 (26)	0 (0)	0 (0)	3 (30)		N/A	N/A	0 (0)		0 (0)	0 (0)
	Asian	3 (16)	1 (25)	0 (0)	3 (30)		N/A	N/A	1 (25)		1 (25)	0 (0)
	Other	1 (5)	0 (0)	0 (0)	0 (0)		N/A	N/A	0 (0)		0 (0)	0 (0)

^a^N/A: not applicable.

^b^N/R: not recorded.

### Intervention

The used immersive reality experience hardware included an Oculus Go (Meta Platforms) all-in-one VR headset and laptop, either of which could be used by children who are hospitalized to see and hear others, and an Insta 360 Pro 360° revolving camera and screen that could be situated in homes or schools for a young person who was absent from that environment to virtually see and move around the environment. In addition, the intervention included a buddy system, whereby a designated individual could communicate with the young person who is hospitalized via text. The young person who is hospitalized would contact the teacher in advance of a scheduled lesson. Cameras were turned on by the teacher at the start of the class and left on until the young person no longer felt like participating. The equipment had no recording capability.

### Data Collection

Following the completion of consent procedures and paper-based outcome measures, for the quantitative portion of this study, young people were given access to immersive reality experience technology for a 6-week period, and then the outcome measures were repeated. We used the following outcome measures: the Short Warwick–Edinburgh Mental Well-Being Scale (SWEMWBS), Social Connectedness Scale, and Social Inclusion Scale. The SWEMWBS is a short version of the 14-item Warwick–Edinburgh Mental Well-Being Scale, which comprises positively worded items measuring different aspects of positive mental health [19,20]. The SWEMWBS is a 7-item scale that asks participants to rate their experience of a range of thoughts and feelings (eg, “I’ve been dealing with problems well”) over the past 2 weeks on a 5-point Likert scale ranging from 1 (*none of the time*) to 5 (*all of the time*). The score is calculated by summing the individual ratings and transforming the total into a metric score using a transformation provided by the scale authors. Scores range from 7 to 35, with higher scores indicating greater positive mental well-being. The original scale has demonstrated good content validity and is correlated with other mental health and well-being measures [19]. The short version has demonstrated similar reliability and validity to the full version (α=.84) and is suitable for use by adolescents [21,22]. The Social Connectedness Scale is a 20-item scale measuring the degree of interpersonal closeness that individuals feel between themselves and other people, both friends and society. Sample items include *I feel disconnected from the world around me* and *I don’t feel related to anyone*. Items are rated from 0 to 6, with higher scores representing a stronger sense of belonging. The scale has been shown to have good internal and test–retest reliability [23,24]. We used an abbreviated version of the scale, comprising the first 8 positively framed items, with a total score of 48. The Social Inclusion Scale is a 22-item scale for measuring social inclusion that has been validated in young adults and contains three subscales for social isolation, relations, and acceptance [25]. We adapted some of the language for use with our adolescent population but did not change any of the actual items.

Qualitative data were collected through separate semistructured interviews, lasting between 30 and 45 minutes each, and were then undertaken by two members of the research team (CF and LAC) with 16 participants, including 4 (25%) young people (3, 75% female and 1, 25% male), 4 (25%) caregivers (all mothers), and 7 teachers (4, 57% female and 1, 43% male), as well as the project custodian. The aim of the interviews was to understand views on the acceptability and usefulness of the immersive reality experience technology. Interviewees reported that the number of engagements (ie, times the young person connected to their classroom via immersive reality experience technology) varied between 3 and 12 sessions. The typical length of each engagement was between 30 and 60 minutes. Engagements were usually shorter when students were in the hospital and received treatment on the day.

### Data Analysis

Quantitative data were analyzed by two members of the research team (HT and HK) using Microsoft Excel (version 16) and IBM SPSS (version 25). Quantitative analyses included basic descriptive statistics (eg, changes in scores on validated scales and demographic characteristics of the sample), and changes in social connectedness, well-being, and social inclusion were evaluated using the nonparametric Wilcoxon signed-rank test. A *P* value of <.05 was taken to indicate statistical significance, and 95% CIs were used to establish the extent of any difference between before and after measures. A sample size of 40 was calculated a priori using Strata (version 15) software to enable detection of changes of 0.5 SD in the primary measure of well-being (using the SWEMWBS) with 80% power. Interviews were audiotaped using a Phillips VoiceTracer digital recorder. The recordings were transcribed, and the transcripts were deidentified by a registered transcriber who had signed a confidentiality agreement with the University of Auckland. To analyze the data, individual transcripts were coded thematically using a 6-step coding process using NVivo 12 software (QSR International) [26]. After transcript familiarization, separate codes were linked to one of three separate case groupings distinguished as either caregiver, teacher, or young people’s reflections on the project. Under each of these groups, the codes were arranged into categories based on the relationships established among them. The initial categories and subcategories were refined by 2 authors (CF and LAC), only including codes in a category if ≥2 interviewees referred to the idea. Any differences were resolved by consensus. All authors were involved in drafting and reviewing the manuscript.

### Ethical Issues

The study received ethical approval from the New Zealand Health and Disability Ethics Committee in December 2018 (reference: 18/NTB/241). Participants were approached via their clinical teams rather than directly by the research team to minimize coercion. Consent for participation was obtained directly for those aged >16 years and via caregivers with participant assent for those aged <16 years. Consent for participation in semistructured interviews by young people, caregivers, and teachers was obtained separately. School principals provided signed consent for their teachers and students to be involved in the trial. Participants were informed that they were free to depart from the trial at any stage. All data were deidentified before analysis and publication.

## Results

### Quantitative Results

At baseline, participants reported moderate levels of well-being, social connectedness, and social inclusion. Following the use of immersive reality experience technology, 70% (7/10) of participants reported improved social inclusion, 80% (8/10) of participants reported improved well-being, and all participants reported improvement in social connectedness. Changes in social connectedness (*P*<.05) were statistically significant, as described in Table 2.

**Table 2:** Changes in social isolation, social connectedness, and well-being following the use of immersive reality experience technology (N=10).

Measures		SWEMWBS^a^		SCS^b^			SIS^c^	
		Before	After	Before	After	Before		After
Values, mean (SD; range)		22.4 (5.2; 17-32)	28.1 (4.5; 20-35)	27.6 (11.2; 10-45)	41.8 (6.9; 29-48)	42.1 (4.8; 34-49)		46 (4.2; 40-54)
Values, mean difference (SD)		N/A^d^	5.7 (4.0)	N/A	14.2 (10.0)	N/A		3.9 (2.8)
**Wilcoxon signed-rank test**								
	Negative ranks	N/A	0^e^	N/A	0^e^	N/A		3^e^
	Positive ranks	N/A	8^f^	N/A	10^f^	N/A		7^f^
	Ties	N/A	2^g^	N/A	0^g^	N/A		0^g^
	Total	N/A	10	N/A	10	N/A		10
	*P* value	N/A	.12	N/A	.01	N/A		.07

^a^SWEMWBS: Short Warwick–Edinburgh Mental Well-being Scale.

^b^SCS: Social Connectedness Scale.

^c^SIS: Social Inclusion Scale.

^d^N/A: not applicable.

^e^Post–immersive reality experience scores lesser than pre–immersive reality experience scores.

^f^Post–immersive reality experience scores greater than pre–immersive reality experience scores.

^g^Post–immersive reality experience scores equal to pre–immersive reality experience scores.

### Qualitative Results

Four major themes were derived from the experiences of young people, caregivers, and teachers involved in the trial:

1. Support for immersive reality experience technology
2. Connecting versus connectedness
3. Choice and connection
4. Setting it up and getting it right

All participant quotes are distinguished by the following: young person, caregiver, and teacher.

### Support for Immersive Reality Experience Technology

Young people in this project echoed the sentiments of those who trialed the equipment assessment in 2018 in terms of their support for the technology [18]. Immersive feelings of *being there* (*in the chosen environment*) and the project as *cool* were still prevalent descriptors about the appeal of the technology by young people:

It feels like you’re kind of there, so I think that was really cool, when you think about it...I was fascinated about the idea, you know? On the headset...you could watch things...there were games and stuff. So, it was pretty cool doing that.

young person 4

I talked to my friends, and they said that it was pretty cool, and they would love to do it.

young person 19

Caregivers also expressed the same enthusiasm for the technology:

Funnily enough at parent interviews lots of parents have said, “hey my daughter had come home” even though she wasn’t in that particular Year 9 maths class, “and talked about this camera. I think it’s amazing that your school has taken this on board!”

teacher 18

Contributing specifically to the appeal of the technology was its ease of setup, as noted by different teachers:

It would take all of about a minute to get going and I didn’t feel that was too time-consuming.

teacher 24

The camera is easy, it was all plugged in, you just had to press the button.

teacher 13

The camera obviously was very unobtrusive, so it created no real difficulties for me in the classroom. It’s just a very small device on a stand that we positioned in the middle of the class...it gave no real concerns about us getting around it.

teacher 18

Barriers to support for the technology in schools were initially encountered in terms of the ethical concerns related to recording children. However, this was resolved swiftly once permissions were obtained, and staff were briefed on how the technology would function:

Other than the fact that the school had to sort permissions and stuff, but once they realized classes won’t be recorded, there were no problems.

teacher 3

Several teachers noted the capacity of the technology in the classroom to generate opportunities for improved understanding about fellow students who were homebound or hospitalized. A teacher recalled a discussion about inclusion and isolation with her students:

The biggest thing is that I saw it as a learning opportunity for the other students in the class. We had some really nice conversations about inclusion...When they see it there, they think, oh is that [name of young person who is home or hospital-bound]? And then my other students would notice the camera, particularly Year 12 or Year 10 and it’s like, “what’s that?” I’d explain to them what it is, and they’re like, “What? So, she still has to do her homework?...Like oh that’s ridiculous, you can’t even escape it in hospital.” I’m like, no—I explained it’s not for learning. They’re like “Oh Wow.” And then they realize the real reason...That opens up conversations about isolation and how fortunate they are to be in school, healthy, with their friends, all that stuff.

teacher 6

All caregivers and teachers acknowledged that they would not hesitate to tell other families about the Patience Project as it helps young people retain a sense of familiarity with the learning environment and their peers. Young people echoed these sentiments, as best exemplified by the following participant:

Everyone who’s in hospital and going through the same thing, just to catch up with your friends and feel like you’re kind of connected in the same way. It’s just good...’cause you don’t wanna miss out on being with your friends

young person 4

### Connecting Versus Connectedness

Although the immersive reality experience technology enabled young people to connect to their chosen environments, connecting did not always result in the sense of connectedness. Caregivers shared mixed perceptions (Textbox 1) on whether the technology had significantly affected the young person’s connectedness to their peers or made or was making their ability to return to school easier. Some caregivers concluded that there was no significant impact in these areas and suggested there might even be an increase in stress because of a sense of obligation to participate. Others described the connections made to the classroom as beneficial to break up the monotony of hospital and recovery days. In a few instances, the same caregiver noted that the connectedness capacity felt a little superficial from their perception but also acknowledged the joy experienced through participating.

**Caregivers’ mixed perceptions of participation benefits for the young person.**

**Participation as beneficial for connectedness**

- “My opinion is that even if you’re [the YP] just there in the lesson it’s better than not being there, because you’ll pick up something rather than nothing.” [caregiver 20]
- “I was in tears, tears of joy rather. Not sadness, joy. Thinking wow, there’s something for kids who really do feel isolated. Because [she] was having a really tough time. And the first time when she connected I think I had the biggest smile on my face, seeing her smile.” [caregiver 5]
- “I guess with the camera, the kids hadn’t forgotten him.” [caregiver 29]
- “I think there’s been one child, one or two children who came and met her at hospital, which means that this connection does help. Because in the past we’ve really never had many people come and visit from school.” [caregiver 5]

**Participation as nonsignificant for connectedness**

- “You know they may have anxiety about, ‘well I’m on this and I'm supposed to be doing this so many times a week. And it’s not happening’. So yeah, I guess in some ways it could add to a bit of stress, feeling like they have to make sure they're on it.” [caregiver 2]
- “I don’t think that it built...any new friendships or anything like that. I don’t think that the camera would have helped him feel like he can fit in again...I think it’s superficial and I also think it’s a huge novelty at the start.” [caregiver 29]
- “I don’t think it’s really made a major significant difference to be honest.” [caregiver 2]
- “She was excited the first time. The second time she was like ‘Mum, it’s boring. Cause English is boring. ’ She was only connecting because she had friends in that class. And the thing is, because the class is still going, you’re not able to have a specific conversation with your friend, right?” [caregiver 5]

Caregivers predominantly held a help-more-than-hinder attitude toward the technology. However, the acknowledgment that some aspects of the experience and the context within which it was engaged might be limiting is an important reminder that technology might interrupt or reduce feelings of isolation but not always incite a sense of connection. Two young people also reiterated the fact that the novelty of participating wears off, as exemplified by the following quote:

I kind of just got used to it. Turned it on, texted me just through text for a bit. And then when class started, they’d get back into it, they would be doing their work and I would be doing nothing much.

young person 19

These connecting experiences were also influenced by a number of factors, including whether the connection was stable, whether the young person’s buddy was reliable, the effectiveness of the interaction loop (ie, between the young person, their buddy, and the teacher), and whether the young person was feeling unwell on the day they chose to connect. Despite these factors, and in light of the insights regarding participation as potentially nonsignificant for connectedness, future adaptions of the project should consider actions to enhance the formation of strong connections, as shared by the following young person:

It would’ve been nice to be able to shift classes and maybe even be able to participate a bit more or something like that. Maybe even be able to have like a virtual worksheet or something that I could do along with the class. Instead of sitting there watching them do the work.

young person 28

The need for more interactive participation was stated by all the young people.

Although not producing a sense of connectedness in all instances, all participants in semistructured interviews acknowledged that using the immersive reality experience technology was beneficial as a means of maintaining a connection with the school environment, which is best represented by the following quote:

I was able to connect with my teachers a bit more. And about who they are a bit and understand what their expectations or something are. They can get to know me and everything too. So, it was much more comforting than showing up to the first day of class and just being shocked because some teacher might yell at you for using the wrong kind of pencil or something like that.

young person 28

Caregivers also saw gaining familiarity with the school environment through the camera as facilitating the return to school:

I think it would help her in the sense that she knows what’s been going on. So, you know you’d hope that she’d be able to slot back in quite easily.

caregiver 2

Building on the idea of maintaining a connection, teachers noted the technology as a good way of easing back into school life after extended periods away through maintenance of connections with peers:

The girls were very engaging with the camera, they would talk to her in the camera, I would call her name out on the role every day. We’d all wave to her...She couldn’t talk back to the girls, but they would say, hey what are you doing? How’s it going? We’re doing this in class. Oh, we’ve got a mufti (casual attire) day today because of da, da, da. All that type of thing and then occasionally she’d message the buddy back on her computer and just say, hey, nice to see everyone, wish I was there, say hello to everyone for me. But instantly there was that connection going backwards and forwards, which was fantastic.

teacher 18

Another teacher noted that education was a secondary benefit of using the technology:

I think some of these kids are so sick, who cares if they’re not doing the work...it’s a connectedness, the feeling of belonging, able to see their peers through that lens, able to feel like they’re part of that classroom again. It might just be a little, you know, half an hour of their day which they feel like a normal teenager.

teacher 18

Caregivers shared similar sentiments about the purpose of the technology to be more about peer connections than education:

It’s not about learning English. It’s more about connecting with your friends and being able to, as I said, go back and find it easier to go back.

caregiver 5

### Choice and Connection

For the young person, connecting and building connectedness was centered on choice (ie, who, what, when, where, and how of their choices).

#### When and Where to Connect?

Timing is crucial for young people wanting to and feeling physically able to connect. Young people predominantly made a choice to connect to the classroom where they could spend the most time (ie, choosing English, as that class was also the young person’s homeroom where they could have informal conversations). However, sometimes the choice of room or timing did not equate to a satisfying participation experience, as noted by the following young person:

It was a very hit or miss sort of deal. Because, of course, if I had social studies in first period, I needed to make sure that I was up, like, by 9 o’clock, get on everything, do all that, especially considering when I first woke up, I would get, yeah, nausea, just a bit of sort of, like, not feeling great. And then you hop into virtual reality and you’re surrounded by all this noise, and it’s just sort of hard, yeah.

young person 28

A caregiver reiterated this young person’s sentiment in describing her daughters’ hit or miss experience:

When you’ve got a sick child it’s actually day by day. So the practicality of it—we’d wake up some days and she would be like yeah, I’m good to go...other days she’d wake up and go no, I’m not getting out of bed today..., it’s one of those things.

caregiver 20

Having increased possibilities for when to connect may foster a sense of control or power over aspects of daily life that have been lost with the illness.

#### How and With Whom to Connect?

Considering the social nature of the Patience Project, it is imperative to understand the different types of dynamics at play, including the fact that there is a buddy system. In particular, it is important to consider how decisions about the buddy system are determined and perceptions of friendships are formed by other students. Some participants described very positive experiences with their buddy, whereas others noted jealousy arising among peers in the classroom, who assumed they should be the designated buddy, with some disturbances arising in the friendships:

At one stage, one was definitely on the outer. But I’m not sure. I wondered if it was because [the buddy] had been chosen as [the young person’s] buddy. I even wondered if there was a little bit of jealousy there, I’ll be honest...I did alert the dean to it just in case she could have a quiet word.

teacher 21

Other disturbances to friendships were also expressed. A young person noted their disappointment after their chosen buddies stopped communicating frequently:

My close friends...didn’t come and I was just really upset about that. I didn’t really wanna talk to all of them then, and I was just really upset because I would always put it in the group chat and be like, “hey guys, you wanna come over?”...My friends that were really nice and wanted to come are on my headset thing. And none of my other friends really knew about it. But when I tried to tell them...it felt like their lives just kept going and they didn’t really care about me.

young person 4

This young person’s experience is an important reminder to not underestimate the fact that when young people have choices with technology, it may come with risks.

#### Why Connect?

All caregivers and young people similarly stressed the need to view the project as noncompulsory, as conveyed in the following statement:

I do recommend it to someone for socialisation. But I would probably stress to them, like, heavily stress to them it’s not compulsory, it’s not for education, it's for socialisation.

young person 28

However, some caregivers and young people conveyed difficulty in engaging out of a sense of obligation because of the opportunity versus genuine motivation on the day:

This is purely from us and not from [project custodian] because I think she goes through so much trouble having it all set up and this camera couriering around the country, and the school setting it up and all of that. I think that you feel “I really should [connect].” Like all this trouble’s happened for me. Not that there’s any pressure at all put on from the Patience Project end. But I think that’s just natural human nature, that if somebody’s done something for you—you want to make the most of it.

caregiver 29

### Setting It Up and Getting It Right

#### Overview

When it comes to enhancing the experience of using the immersive reality experience technology to connect and build connectedness, participants asserted the need to get it fully right from the initial setup. Otherwise, it may become *a bridge too far*. A young person’s ability to engage was conveyed as hinging on three important factors:

1. The technology must work well every time.
  Participants in all groups recalled technical glitches in terms of noise, connection difficulties, and inability to see the board or teacher effectively at some points.
2. To maximize the use of technology, it needs to be easily movable.
  This factor builds on the findings previously mentioned and aims to put power back into the hands of the young person, allowing them to facilitate choice over the environment for the camera. Teachers communicated that the increased mobility of the camera might enhance the educational capacity of the technology.
3. The ease of use for all parties involved is imperative.
  Participants indicated that if they had to struggle with any part of the process of connecting, they would give up and consider trying another time.

Participants across all groups suggested ideas concerning these three factors, which they viewed as potentially enhancing the experience from the start, each fitting within one of the five categories:

1. Connecting participating teachers
2. Formation of bonds
3. Mock sessions before participation
4. Device mobility
5. Education about virtual connections

#### Connecting Participating Teachers

This involves a channel to share specifics of the process and any inclusive activities to engage students on the other side of the camera.

#### Formation of Bonds

Bonds with the teacher and a buddy were central to the project’s success and moved it from the aforementioned *a bridge too far*. From the young person’s perspective, when a buddy was unreliable, their connecting experience was compromised. Young people reported mixed experiences in this regard:

Because my buddy just really wasn’t in touch at all. And then I couldn’t find when my class.

young person 1

versus

She turned it on and she turned it off. She was fun. She was a lot of fun in the class, so she could text me and I could text her.

young person 19

Two caregivers suggested that it might be best for the teacher to choose someone they perceive would be a reliable buddy so as to not risk existing friendships and create opportunities to build new connections. Caregivers indicated that an individual such as the project custodian is imperative to young people’s engagement and ensuring that buddies and teachers fulfill their roles. Both caregivers and young people were appreciative of the participating teachers, specifically those who embraced a new way for young people to connect to the classroom. Participants deemed it essential to have a competent teacher who will make the most of the technology and use the experience to educate other students about illness and isolation. Gauging this enthusiasm early may be a good indication of the type of interaction loop that will arise between the young person, the buddy, and their teacher.

#### Mock Sessions Before Participation

Having an understanding of what the young person could see on the other side of the camera was described as an important consideration for teachers to understand the experience more holistically so that they can serve the students’ learning and connecting needs more effectively (ie, is board work clear? Is the camera close enough to see the content?). The idea of participating in mock sessions was also proposed as a support to help the chosen buddy grasp the importance of the session.

#### Device Mobility

It was unanimously suggested that if the device were easily mobile, the number of engagements would be far greater, and the student’s experience would perhaps be increasingly meaningful.

#### Education About Virtual Connection

Participants referred to the need for education about the project and education about virtual inclusion initiatives. A teacher described their future tips for class education as being central to getting it right from the beginning:

...the teacher needs to spend the time explaining the meaning behind it, why has it been designed, if you were in the students shoes do you think it would be helpful for you? Show the clip, Ben explaining his reasons for developing it...when the kids have full context they engage with it, they’re completely on your side, they engage with the participant and then I think they get 100 times more out of it. Being totally honest and upfront with the class from the beginning I think gives you the best possible outcome.

teacher 18

Two teachers described how in parent–teacher interviews, caregivers said, “I think it’s amazing that your school’s taken this onboard” after expressing their child had come home describing the technology and its purpose. Young people also highlighted the need for further training on how to use the technology:

There was a bit of a problem with the VR set...because I didn’t know how to make it 360, I couldn’t find the setting. So it was just on a big screen. I was on my laptop. So, yeah. But it was fine other than that.

young person 19

Both teachers and caregivers noted initial concerns about who else would see into the classroom, whether the sessions were being recorded, and whether the teacher was being judged by onlookers. The Patience Project was designed for the child’s eyes only, and after speaking with the project custodian, teachers and students in the classroom felt assured that privacy would be upheld.

Adding to a need for education, encouraging the heads of schools to use the project as a tool to bridge unmet needs was also described by a caregiver. Helping teachers and heads of school move beyond *the too hard basket* mentality when it comes to children who are home and hospital bound was a significant concern of a few caregivers who struggled to find support for their children upon initial diagnosis. Thus, the Patience Project helped with connection to educational and social environments, even if only for familiarity purposes—a need not previously met, as described by a caregiver:

About October she was feeling better and she goes “mum, can you get me some work?”...I contacted her teacher and no one would get back to us. It was in the too hard basket for them. I asked, “Can these kids not Skype into their classrooms? We Skype all the time and you're telling me we can’t do this?”

caregiver 2

Educating the staff (head teachers to classroom teachers) was also noted as a way of fostering supportive environments for the immersive reality experience technology for schools:

I spoke at a staff afterschool meeting, talked about it and showed how it worked...did some photos, did a little video clip of how it works in my general day to day class room and explained it to our staff of 120. And at the end they were...blown away! I guess, by the technology and the opportunity that students like [she] could get by having that camera in the class.

teacher 18

These education sessions often turned into curiosity about the technology, as another teacher noted the following:

I obviously briefed all the teachers; told them about the camera in my class and the reason for it etcetera. They thought it was a worthwhile initiative and some came round to have a look at how it worked.

teacher 3

Aside from one teacher who was respecting the privacy of the participant, all participants described sharing with staff and students as a positive experience, and all stated that they would happily be involved again.

## Discussion

### Principal Findings

Our results provide valuable information regarding the acceptability of immersive reality experience technology and what might be required for its successful implementation. The technology appealed to many of our trial participants, and there was an acknowledgment of its potential to facilitate learning and ease the transition back to normal life, particularly school following an illness. Our findings also provide a preliminary indication of its effectiveness at improving well-being and social connectedness and, to a lesser extent, improving social inclusion and disrupting social isolation for young people with long-term physical conditions.

A handful of other devices and web-based and text-, audio-, and video-based technologies have been trialed over the past couple of decades to connect children who are hospitalized with schools and meet their academic and social needs in international contexts outside New Zealand [27]. These include a communication app for young people with cystic fibrosis [28], an ambient technology-based orb in the classroom [29], and the Presence app [30]. To date, no studies have used virtual or immersive reality. Most previously studied interventions demonstrated similar qualitative acceptability to that of immersive reality experience technology [14,31]. Only one open trial of the 2-way, web-based Bednet tool [32] demonstrated improved social connectedness using a Likert scale, and a nonrandomized trial of a CareRabbit robot that helps children stay in touch with family and friends [33] has demonstrated nonsignificant differences in well-being between groups. Thus, our study is the first trial of a hospital to school communication system to demonstrate improvements in both social connectedness and well-being.

A number of participants in our trial experienced personal, health-related, technological challenges and school-related barriers to its use. Some simply did not find the technology engaging enough to continue using it. Others experienced challenges in getting the equipment to work. Duration and frequency of use were often related to users’ state of health or treatment schedules, with greater use on days when they were feeling well or not attending medical appointments. Acceptance of cameras in classrooms, knowing how to use them, and socializing classroom buddies and fellow students also proved difficult for some schools. These issues have all been experienced by the developers of similar interventions [27] and, rather than being reasons for their disuse, are probably key barriers to target during implementations. They are also issues to consider during the design of a more definitive RCT. Allowing greater time, sourcing participants from a larger catchment, and engaging schools in advance of participant recruitment would be useful.

Young people’s sense of connectedness appeared to be dependent on everyone else’s connection and ability to foster connectedness (eg, the buddy connecting, friends continuing to engage with them through the device, family or whanau communicating with teachers when a child is unwell, and teachers communicating classroom activities and checking in via the device). For some, the social connectedness they experienced was superficial; however, they still embraced the moments of interrupted isolation the technology offered. For others, it worked exceptionally well, indicating that all the dependent factors functioned in cohesion. It was reassuring that most caregivers and teachers supported the use of the immersive reality experience technology. Although some caregivers were focused on the educational benefits of connection between hospitals and schools, most appreciated the value of social connectedness for their children’s well-being. Teachers were also positive about the child’s right to inclusivity and the formation of new, and maintenance of old, friendships between students in the classroom and young people who are hospitalized. This may be attributed to the fact that they were witnessing (at least in part) what the young person was seeing on the other side of the camera in terms of peers in the classroom speaking to the camera, asking questions about the situation, and seeing the 2-way communication loop (ie, texting) between the buddy and young person occur. Effective education regarding the purpose of the trial and training in how to use immersive reality experience equipment were key parts of this process.

VR-based technology is not new. It has been shown to be useful for distraction, pain reduction, and relaxation during the treatment of children and young people who are hospitalized and has improved in quality over the past decade [34,35]. Illness or treatment-related nausea reportedly detracts a subset from fully engaging with the immersive reality experience technology. A future trial that includes VR and non-VR arms would help clarify the additive value of VR headsets. Having a reliable *buddy* at the other end of the connection was a more relevant issue for most participants. Being able to connect in a flexible manner, including being able to choose between the use of VR and non-VR methods, also probably helped to foster a sense of control or power over aspects of daily life that have been lost with illness. The reliability of technology appeared to be especially important for participants, with connection difficulties and inability to see the teacher or board and inability to move the camera sometimes proving to be *a bridge too far*. Although the COVID-19 pandemic significantly affected recruitment, it may also have normalized children’s access to educational environments through virtual media. Evident from the increasing openness of teachers to immersive reality experience technology toward the end of the trial, this phenomenon deserves greater investigation.

The strengths of this trial include participation by young people with a range of long-term physical conditions, the collection of both quantitative and qualitative data with which to better understand the experience of individuals using immersive reality experience technology, and triangulation of participant views with those of their caregivers and teachers. Low recruitment because of COVID-19–related restrictions in access to patients who are hospitalized and the consequent absence of any participants aged 17 to 18 years were the main limitations of this trial, as was the absence of any qualitative data from participants who did not use the intervention and those who were too unwell or elected not to be interviewed, which might have provided less favorable perspectives. Nonetheless, we were pleased to observe qualitative evidence of acceptability and improvement in all quantitative outcomes. The generalization of trial results to other settings cannot be assumed from our findings, nor can effectiveness and acceptability to individuals from different cultural backgrounds. A larger trial is needed to confirm our preliminary quantitative results. It would also be useful to collect objective data regarding the actual time spent using the immersive reality experience technology. A more in-depth analysis of hospital and school-related factors affecting engagement and setup; the impact of COVID-19 on openness to virtual communication between hospitals and schools; the use of e-mentors, as suggested by Ellis et al [36]; and the value of liaison workers in schools (eg, health school staff and health school teachers) [37] would be useful to augment the effective use of immersive reality experience. Considering feedback to date, immersive reality experience technology is being adapted and integrated into lower cost, multimodal communication by the Patience Project Charitable Trust. This should improve its portability and applicability to a greater number of users.

### Conclusions

This trial demonstrates that immersive reality experience technology has the potential to improve the well-being, social connectedness, and social inclusion of New Zealand children and young people who are hospitalized. It also provides valuable information regarding the feasibility of a more definitive RCT. With some technological modifications and simplified implementation, immersive reality experience and related communication technology could become part of standard care and support children and young people who are hospitalized in New Zealand and elsewhere to sustain family and peer cohesion, experience fewer psychological problems, and more easily return to school and normal life following completion of treatment.

## Abbreviations

RCT: randomized controlled trial
SWEMWBS: Short Warwick–Edinburgh Mental Well-Being Scale
VR: virtual reality

## References

[1] Crengle S, Clark TC, Robinson E, Bullen P, Dyson B, Denny S, Fleming T, Fortune S, Peiris-John R, Utter J, Rossen F, Sheridan J, Teevale T, The Adolescent Health Research Group (2013). The Health and Wellbeing of Māori New Zealand Secondary School Students in 2012. Te Ara Whakapiki Taitamariki: Youth’12.

[2] Gilmour M, Hopkins L, Meyers G, Nell C, Stafford N (2015). School Connection for Seriously Sick Kids. Who Are They, How Do We Know What Works, and Whose Job Is It?.

[3] Eiser C (2018). Effects of chronic illness on children and their families. Adv Psychiatr Treat.

[4] Park C, Majeed A, Gill H, Tamura J, Ho RC, Mansur RB, Nasri F, Lee Y, Rosenblat JD, Wong E, McIntyre RS (2020). The effect of loneliness on distinct health outcomes: a comprehensive review and meta-analysis. Psychiatry Res.

[5] Loades ME, Chatburn E, Higson-Sweeney N, Reynolds S, Shafran R, Brigden A, Linney C, McManus MN, Borwick C, Crawley E (2020). Rapid systematic review: the impact of social isolation and loneliness on the mental health of children and adolescents in the context of COVID-19. J Am Acad Child Adolesc Psychiatry.

[6] Muntaner JJ, Forteza D, Salom M (2014). The inclusion of students with chronic diseases in regular schools. Procedia Soc Behav Sci.

[7] Mitchell W, Clarke S, Sloper P (2005). Survey of psychosocial support provided by UK paediatric oncology centres. Arch Dis Child.

[8] Anderson M, Elliott EJ, Zurynski YA (2013). Australian families living with rare disease: experiences of diagnosis, health services use and needs for psychosocial support. Orphanet J Rare Dis.

[9] Lal S, Adair CE (2014). E-mental health: a rapid review of the literature. Psychiatr Serv.

[10] Thabrew H, Stasiak K, Hetrick S, Wong S, Huss J, Merry S (2018). E-Health interventions for anxiety and depression in children and adolescents with long-term physical conditions. Cochrane Database Syst Rev.

[11] Flavián C, Ibáñez-Sánchez S, Orús C (2019). The impact of virtual, augmented and mixed reality technologies on the customer experience. J Bus Res.

[12] Bowler DE, Buyung-Ali LM, Knight TM, Pullin AS (2010). A systematic review of evidence for the added benefits to health of exposure to natural environments. BMC Public Health.

[13] Coon JT, Boddy K, Stein K, Whear R, Barton J, Depledge MH (2011). Does participating in physical activity in outdoor natural environments have a greater effect on physical and mental wellbeing than physical activity indoors? A systematic review. Environ Sci Technol.

[14] Hopkins L, Wadley G, Vetere F, Fong M, Green J (2014). Utilising technology to connect the hospital and the classroom: maintaining connections using tablet computers and a ‘Presence’ App. Aus J Edu.

[15] Soares N, Kay JC, Craven G (2017). Mobile robotic telepresence solutions for the education of hospitalized children. Perspect Health Inf Manag.

[16] Beeman RY, Henderson CJ (2015). Video-conferencing technology brings a homebound middle grades student to the classroom. Middle School J.

[17] Lombaert E, Veevaete P, Schuurman D, Hauttekeete L, Valcke M (2006). A special tool for special children: creating an ICT tool to fulfil the educational and social needs of long-term or chronic sick children. Current Developments in Technology-Assisted Education.

[18] Chubb LA, Fouché CB, Agee M, Thompson A (2021). ‘Being there’: technology to reduce isolation for young people with significant illness. Int J Inclus Edu.

[19] Tennant R, Hiller L, Fishwick R, Platt S, Joseph S, Weich S, Parkinson J, Secker J, Stewart-Brown S (2007). The Warwick-Edinburgh Mental Well-being Scale (WEMWBS): development and UK validation. Health Qual Life Outcomes.

[20] Ng Fat L, Scholes S, Boniface S, Mindell J, Stewart-Brown S (2017). Evaluating and establishing national norms for mental wellbeing using the short Warwick-Edinburgh Mental Well-being Scale (SWEMWBS): findings from the Health Survey for England. Qual Life Res.

[21] McKay MT, Andretta JR (2017). Evidence for the psychometric validity, internal consistency, and measurement invariance of Warwick Edinburgh mental well-being scale scores in Scottish and Irish adolescents. Psychiatry Res.

[22] Ringdal R, Eilertsen MB, Bjørnsen HN, Espnes GA, Moksnes UK (2018). Validation of two versions of the Warwick-Edinburgh Mental Well-Being Scale among Norwegian adolescents. Scand J Public Health.

[23] Lee RM, Robbins SB (1995). Measuring belongingness: The Social Connectedness and the Social Assurance scales. J Counsel Psychol.

[24] Lee RM, Robbins SB (1998). The relationship between social connectedness and anxiety, self-esteem, and social identity. J Counsel Psychol.

[25] Wilson C, Secker J (2015). Validation of the Social Inclusion Scale with students. Soc Incl.

[26] Braun V, Clarke V (2013). Successful Qualitative Research: A Practical Guide for Beginners.

[27] Maor D, Mitchem K (2015). Can technologies make a difference for hospitalized youth: findings from research. J Comput Assist Learn.

[28] Francis J, Cross D, Schultz A, Armstrong D, Nguyen R, Branch-Smith C (2020). Developing a smartphone application to support social connectedness and wellbeing in young people with cystic fibrosis. J Cyst Fibros.

[29] Vetere F, Green J, Nisselle A, Thu Dang X, Zazryn T, Peng Deng P (2012). Inclusion during school absence: using ambient technology to create a classroom presence for hospitalised children. Telecommun J Aus.

[30] Wadley G, Vetere F, Hopkins L, Green J, Kulik L (2014). Exploring ambient technology for connecting hospitalised children with school and home. Int J Hum-Comput Stud.

[31] Maor D, Mitchem K (2018). Hospitalized adolescents’ use of mobile technologies for learning, communication, and well-being. J Adolesc Res.

[32] Zhu C, Van Winkel L (2014). Using an ICT tool as a solution for the educational and social needs of long-term sick adolescents. Technol Pedag Edu.

[33] Blom S, Boere-Boonekamp MM, Stegwee RA (2011). Social connectedness through ICT and the influence on wellbeing: the case of the CareRabbit. Stud Health Technol Inform.

[34] Yap KY, Koh DW, Lee VS, Wong LL (2020). Use of virtual reality in the supportive care management of paediatric patients with cancer. Lancet Child Adolesc Health.

[35] Tennant M, Youssef GJ, McGillivray J, Clark T, McMillan L, McCarthy MC (2020). Exploring the use of immersive virtual reality to enhance psychological well-being in pediatric oncology: a pilot randomized controlled trial. Eur J Oncol Nurs.

[36] Ellis SJ, Drew D, Wakefield CE, Saikal SL, Punch D, Cohn RJ (2013). Results of a nurse-led intervention: connecting pediatric cancer patients from the hospital to the school using videoconferencing technologies. J Pediatr Oncol Nurs.

[37] Hopkins L, Green J, Henry J, Edwards B, Wong S (2013). Staying engaged: the role of teachers and schools in keeping young people with health conditions engaged in education. Aust Educ Res.

